# Stimulated migration and penetration of vascular endothelial cells into poly (L-lactic acid) scaffolds under flow conditions

**DOI:** 10.1186/2055-7124-18-7

**Published:** 2014-06-13

**Authors:** Min-Ah Koo, Jae Kyeong Kang, Mi Hee Lee, Hyok Jin Seo, Byeong-Ju Kwon, Kyung Eun You, Min Sung Kim, Dohyun Kim, Jong-Chul Park

**Affiliations:** Cellbiocontrol Laboratory, Department of Medical Engineering, Yonsei University College of Medicine, 134 Shinchon-dong, Seodaemun-gu, Seoul, Korea; Brain Korea PLUS 21 Project for Medical Science, Yonsei University College of Medicine, 134 Shinchon-dong, Seodaemun-gu, Seoul, Korea

**Keywords:** Vascular Endothelial cells, Cell migration, Fluid shear stress, Parallel plate chamber, Polymer scaffolds

## Abstract

**Background:**

The initial procedure of the development of engineered tissues is cell seeding into three-dimensional polymer scaffolds. However, it is hard to make the cells invade into scaffold due to the characteristic of pore and material. Electrospun poly (L-lactic acid) scaffold and flow perfusion system were used to overcome these seeding problems.

**Results:**

Before starting the experiment, we set up the parallel plate chamber system to observe endothelial cell migration under flow condition. In individual cell migration model, human umbilical endothelial cells started to migrate in the direction of flow at 8 dyne/cm^2^ and we observed the cytoskeleton alignment at 8 dyne/cm^2^.

This study has demonstrated the possibility to evaluate and analyze cell migration using the parallel plate chamber system and we may predict *in vivo* cell migration under flow condition based on these results. Also the flow perfusion system was established for the effective cell seeding into at three dimensional scaffolds. Moreover, shear stress induced by flow can enhance cell migration into PLLA scaffold that is in the form of cotton.

**Conclusions:**

Result indicated that cell penetration was achieved under flow condition better and more than under static condition throughout the matrix.

## Background

Tissue engineering is a promising technology that applies the principles of biology and engineering to the development of functional substitutes for injured tissue [1]. One method to tissue engineering is to isolate cells, culture the cells *in vitro*, and seed them into an artificial structure that is able to support three dimensional (3D) tissue formations. This 3D structure is called scaffold. It is known that a porous scaffold is required to allow cell seeding or migration throughout the pore. Therefore, pore size is critical for tissue development and determines the inner surface area for cell attachment [2]. In those processes, cell is seeded to form the 3D tissue-like structures as the first step to make homogeneous tissue grafts.

Tissues created by 3D porous scaffold formed by cells *in vitro* commonly result in an inhomogeneous formation. This kind of inhomogeneous formation may prevent tissues from performing the appropriate functions as the mechanical features and improving the graft properly in the body [3]. 3D scaffold that cells seeded homogeneously will develop a homogeneous graft with a uniform distribution of cells and extra cellular matrix. However, it is technically difficult to seed the cells to scaffold in the same cell distribution. One reason is insufficient migration into the scaffolds because of pore size and material [4]. Therefore, it is necessary to consider the techniques of cell seeding into scaffolds for the optimized *in vitro* cultivation of tissue-like structures.

A variety of bioreactor systems have been considered such as the spinner flask [5], perfused continuously through glass columns [6] and the rotational bioreactor [7] to maintain 3D tissue-engineered constructs and uniformity of cell distribution in scaffold [8]. Fluid-shear forces were produced when flow-perfusion bioreactor was used. Using the flow-perfusion bioreactor, we could generate shear forces and supply enough nutrients to the inner area of the scaffold during culture.

The chemical and physical factors in the vascular system regulate endothelial cells (ECs) migration by different mechanisms. One of these mechanisms is mechanotaxis that induces directional migration in response to mechanical forces [9]. *In vivo*, ECs are continually exposed to fluid shear stress, the tangential component of hemodynamic force because of blood flow. Shear stress has been found to remodel ECs monolayer that induces the mechanism of the signal transduction and gene expression [10] and increase stress fibers [11]. While shear stress is applied on the luminal surface of ECs, the mechanical–chemical signaling can be transmitted throughout the cell and to cell extracellular matrix (ECM) adhesions on the abluminal surface of ECs. There is accumulating evidence suggesting that fluid shear stress can modulate each step of the migration process, including the extension of the leading edge, adhesion to the matrix, and release of adhesions at the rear [9]. The rate and extent of endothelial migration onto a prosthetic material vascular stent surface are influenced by the level and direction of flow-related wall shear stress [6, 12], but the kinetics and molecular mechanism of EC migration in response to shear stress remain to be determined. The purposes of this study are to evaluate effects of shear stress on endothelial cells *in vitro* and to analyze the migration of ECs. To investigate the migration of ECs, we utilized a parallel plate flow chamber system to apply a shear stress to ECs grown on a gelatin coated coverslip. Also, immunostaining was carried out to confirm the actin cytoskeleton alignment. Based on these results, finally we tested the hypothesis that cells penetrate into scaffold in response of flow for uniform cell distribution when cultured under direct flow perfusion.

## Methods

### Cells and cell cultures

Human umbilical vein endothelial cells (HUVEC) were purchased from Cambrex Bio Science Walkersville. These cells were cultured in Endothelial basal medium-2 (EBM-2, Lonza, Walkersville, MD, USA) supplemented with 2% fetal bovine serum (FBS, Lonza) and endothelial cell growth factors (Lonza, Hydrocortisone 0.2 ml, hFGF-B 2 ml, VEGF 0.5 ml, R3-IGF-1 0.5 ml, Ascorbic acid 0.5 ml, hEGF 0.5 ml, GA-1000 0.5 ml, Heparin 0.5 ml). Before cells were seeded, 18 mm round coverslips (Fisherbrand, Leicestershire, United Kingdom) were coated with 2% gelatin (Gelatin from porcine skin Type A, sigma). Before using the coverslip, it was dipped into 100% ethanol and flame sterilized. 500 μl of 2% gelatin solution in phosphate buffered saline (PBS) was added to 18 mm coverslips and incubated for 2 hours at 37°C. After 2 hours, 2% gelatin solution was suctioned and dried in air. For individual cell migration model (nonconfluent), 8 × 10^3^ cells in 600 μl were plated on the coverslip at 30% confluency. The cells were plated onto gelatin-coated coverslips and were incubated for 24 hours before exposure to flow. HUVEC were studied before passage 10 in all experiments.

### Parallel plate chamber system

**Figure 1:**
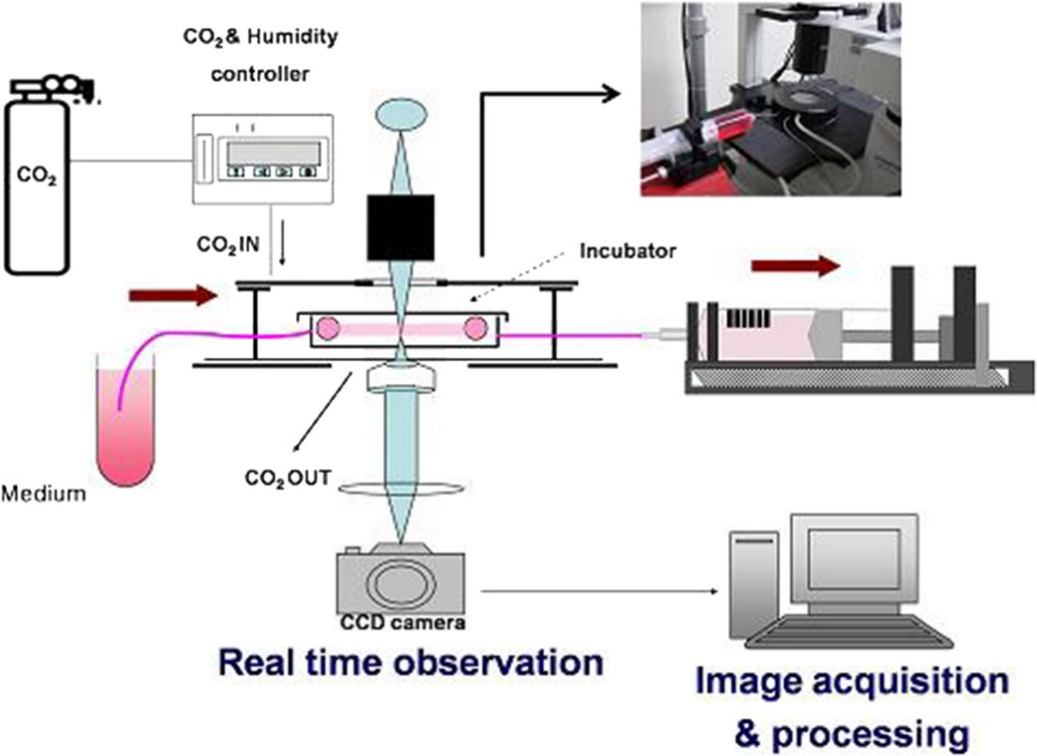
**The parallel plate chamber system to apply shear stress to cells.** Schematic diagram of the parallel-plate flow chambers in a flow system for the evaluation of the cell migration.

We used the parallel plate chamber system (Figure 1) to apply shear stress to HUVEC. The parallel plate chamber system consisted of two parts, incubator system installed with the microscope to observe live cells and the flow chamber to apply shear stress to the cells. The incubator was regulated by temperature and gas composition controlling program (CCP ver. 3.8) under proper environment for cell (5% CO_2_, 37°C). The flow chamber was made up by main body with inlet and outlet for tubing (inner diameter, 2 mm), bottom plate and silicon gasket. Gelatin-coated coverslip seeding HUVEC was mounted on the bottom plate and put the main body and the silicon gasket (200 μm in height, 2 mm in width) together. Medium was taken out at least for 1 hour before starting experiments to prevent bubbles. The shear stress (dyne/cm^2^) was calculated by this equation.

Q is the volumetric flow rate (ml/s), μ is the viscosity of the medium (dynes/cm^2^), W is the gasket width (cm) and h is the gasket height (cm) [13]. It was known that physiological levels of venous and arterial shear stresses are 1–5 and 6–40 dynes/cm^2^, respectively [12]. Thus, we selected 4 and 8 dyne/cm^2^ in the physiological level of shear stress. It caused cell damage to apply shear stress of 10 dyne/cm^2^ and over to the cells.

### Image acquisition

The cells were cultured in the incubator placed on the microscope stage and cell images were recorded every 5 minutes for 8 hours by the change-coupled device (CCD) camera (Electric Biomedical Co. Ltd., Osaka, Japan) attached to the inverted microscope (Olympus Optical Co. Ltd., Tokyo, Japan). Images were conveyed directly from a frame grabber to computer storage using Tomoro image capture program and memorized them as JPEG image files.

### Cell tracking and evaluation of cell migration

For data analysis, captured images were imported into ImageJ (ImageJ 1.37v by W. Rasband, National Institutes of Health, Baltimore, Md). Image analysis was carried out by manual tracking and chemotaxis and migration tool plug-in (v. 1.01, distributed by ibidi GmbH, Mnchen, Germany) in ImageJ software. We obtained the datasets of XY coordinates by using manual tracking. Then, these datasets were imported into chemotaxis and migration tool plug-in. The tool computed the cell migration speed, directionality and X forward migration index (X_FMI_) of HUVEC and plotted cell migration pathway. The migration speed was calculated as an accumulated distance of the cell divided by time. The directionality of the cell was defined as Euclidean distance divided by accumulated distance. The Euclidean distance means the straight-line distance between the start point and the end point. The closer the directionality was to 1, the straighter the cell moved. The X_FMI_ of the cell was defined as an X_FMI_ divided by accumulated distance. Cells undergoing division, death, or migration outside the field of view were excluded from the analysis.

### PLLA scaffold

We were provided with the scaffold from Ehwa Womans University. Poly (L-lactic acid) (PLLA) (intrinsic viscosity 0.63 dl/g, Mw = 2.5 × 10^5^ g/mol) was provided by Purac Biochem (Gorinchem, Netherlands). Dichloromethane (MC) and acetone were purchased from Duksan Chemicals Co. (Seoul, Korea). In brief, 8% w/v PLLA solutions were prepared with the solvent mixture composed of MC and acetone (90:10 v/v). The polymer solution was poured into a 10-mL glass syringe, attached to a 25-gauge blunt end needle. A syringe pump was set at a volume flow rate of 0.1 ml/min. The distance between the needle tip and the collector was 15 cm. The electrospinning process was carried out in a sterile environment at high voltage. A voltage between 8 and 20 kV was used for all solutions. Prior to usage, the electrospun scaffolds were dried for three days under a vacuum at 70°C to remove the solvents.

### The flow perfusion system

**Figure 2:**
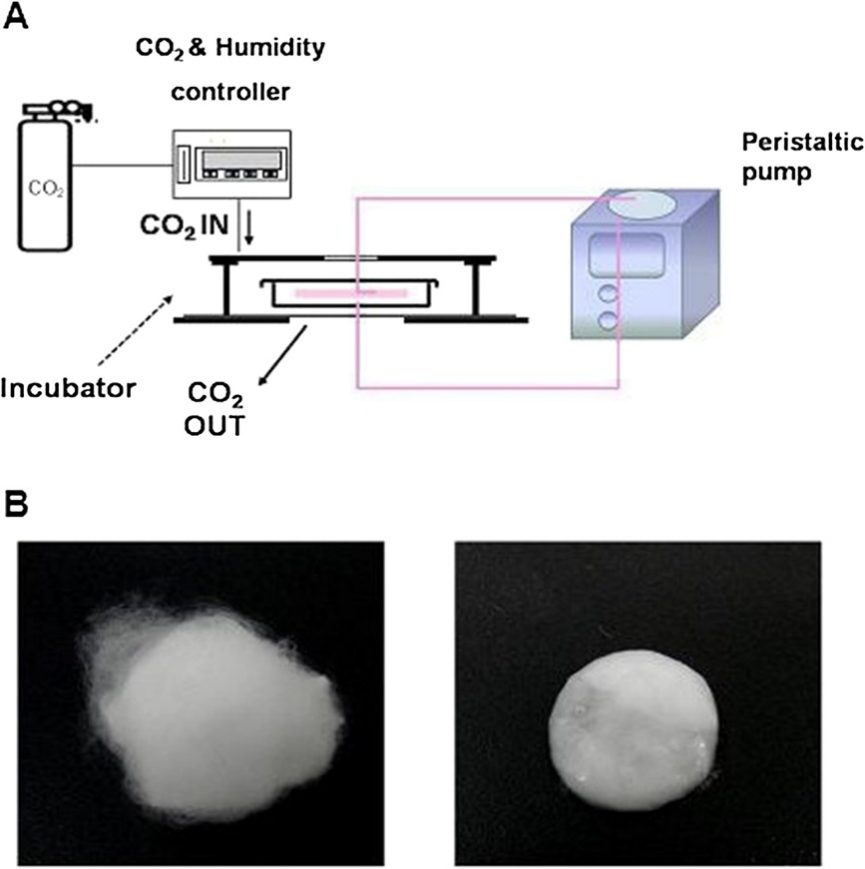
**HUVEC migration into scaffold in response of flow. (A)** Schematic diagram of the flow perfusion system for the evaluation of the cell migration into the scaffold. **(B)** Microfibrous PLLA scaffold. Microfibrous scaffolds of weigh 80 mg were prepared (left) and each of these was placed in a separate well of a 24 well tissue-culture plate for cell seeding (right).

We used the peristaltic pump that produces 500 ml/hour to circulate the medium (Figure 2A). The chamber was tapered to ensure flow from the outer edges of the scaffold as well as the center to the exit port of the chamber. Screw caps were fitted with O-rings for a tight seal and prevention of leakage. The peristaltic pump pulled medium from the reservoir and provided it to the chamber including cell-seeded scaffold via 6 mm inner diameter silicone tubing. Equipment was sterilized by steam autoclave (tubing, chamber). The apparatus was assembled under sterile conditions in a laminar flow biosafety cleanbench. In order to incubate the cells in the chamber, a CO_2_ mini-incubator (150 × 130 × 40 mm) was designed and fabricated with a double-layered acrylic plate. The mini-incubator was connected with a CO_2_ gas mixing system (FC-5, Live cell instrument Inc., Seoul, Korea) and supplied 5% CO_2_.

### Observation of cells distribution in scaffold

After 4 hours seeding the cells, the scaffolds were washed 2 times with phosphate buffered saline (PBS) and the cells were fixed with pre-cooled (−20°C) 70% ethanol for 5 minutes. Then, the cells were stained with propidium iodide (Sigma, Steinheim, Germany). The migration of cells into the scaffold was calculated by a confocal microscope (LSM 510, Carl Zeiss Micro Imaging Inc., North America), using horizontal and vertical sections through the scaffolds every 10 μm.

### Immunostaining

After applying shear stress for 30 minutes and 5 hours, actin cytoskeleton was visualized by immunostaining. Each step for immunostaining was as following. Cells were fixed with 3.7% paraformaldehyde for 15 minutes at room temperature and were washed two times with PBS. Cells were permeabilized with 0.25% Triton X-100 in PBS for 5 minutes at room temperature and rinsed 3 times with PBS. Nonspecific bindings to cells were blocked with 1% bovine serum albumin (BSA) for 30 minutes at room temperature. In dark, they were treated with Alexa (488)-conjugated phalloidin (5 U/ml, Invitrogen) for actin cytoskeleton staining for 30 minutes at room temperature. The monolayers were mounted under a coverslip with aqueous mounting medium (Dako Faramounts, Dako North America Inc., CA, USA) and were observed by a fluorescence inverted microscope.

### Statistical analysis

All statistical analyses were completed with SPSS Software version 12.0 (SPSS, Chicago, IL, USA). Non-normal distributions in the data were not allowed to use Analysis of variance (ANOVA) and t-tests. Therefore, comparisons between groups were carried out using the nonparametric Kruskal-Wallis test. Comparisons between subgroups used the Mann–Whitney U test with Bonferroni correction for multiple comparisons, thus yielding statistical significance if *p < 0.0167*. All data were presented as mean values and standard deviation (SD).

## Results

### Movement of HUVEC in accordance with the shear stress in the individual cell migration model

We characterized the migration speed of the ECs under static and flow conditions. Sub-confluent HUVEC were plated on gelatin-coated cover slip and were then kept as static controls or subjected to shear stress at 4 and 8 dyne/cm^2^. Cell movement was monitored by time-lapse microscopy and time-lapse images were tracked every 5 minutes for 7 hours after cell seeding to evaluate the migration speed and the directionality of cells. The closer the directionality was to 1, the straighter the cell moved.

**Figure 3:**
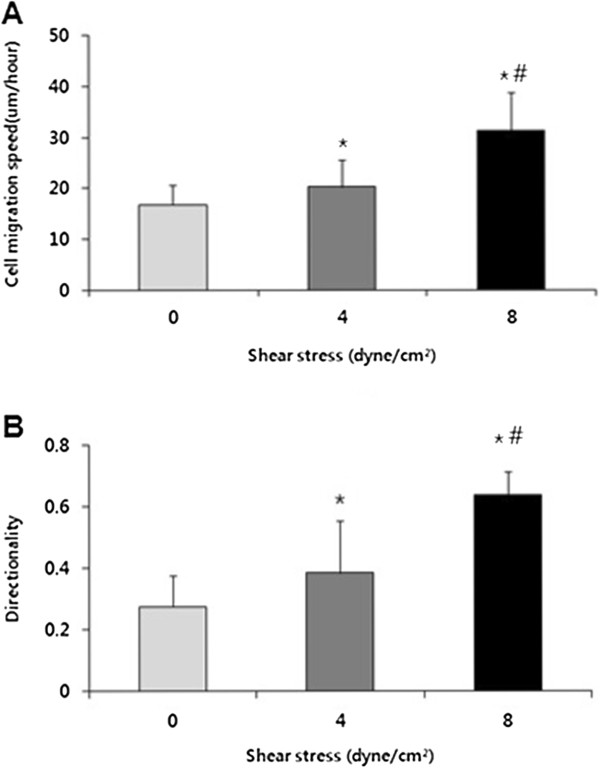
**Movement of HUVEC in accordance with the shear stress in individual cell migration model. (A)** The migration speed of HUVEC. **(B)** The directionality of HUVEC.

The application of shear stress significantly increased the cell migration speed (Figure 3A). In case of the directionality, HUVEC moved more straightly under flow condition compared to the static condition (Figure 3B).

### Directional migration of HUVEC in individual cell migration model

To assess the effect of shear stress has on x directional migration of HUVEC, after tracking the cell movement using manual tracking in ImageJ, x directional migration was calculated as the ratio of the net distance that the cell migrated in the forward direction to the total migration length that the cell traveled.

**Figure 4:**
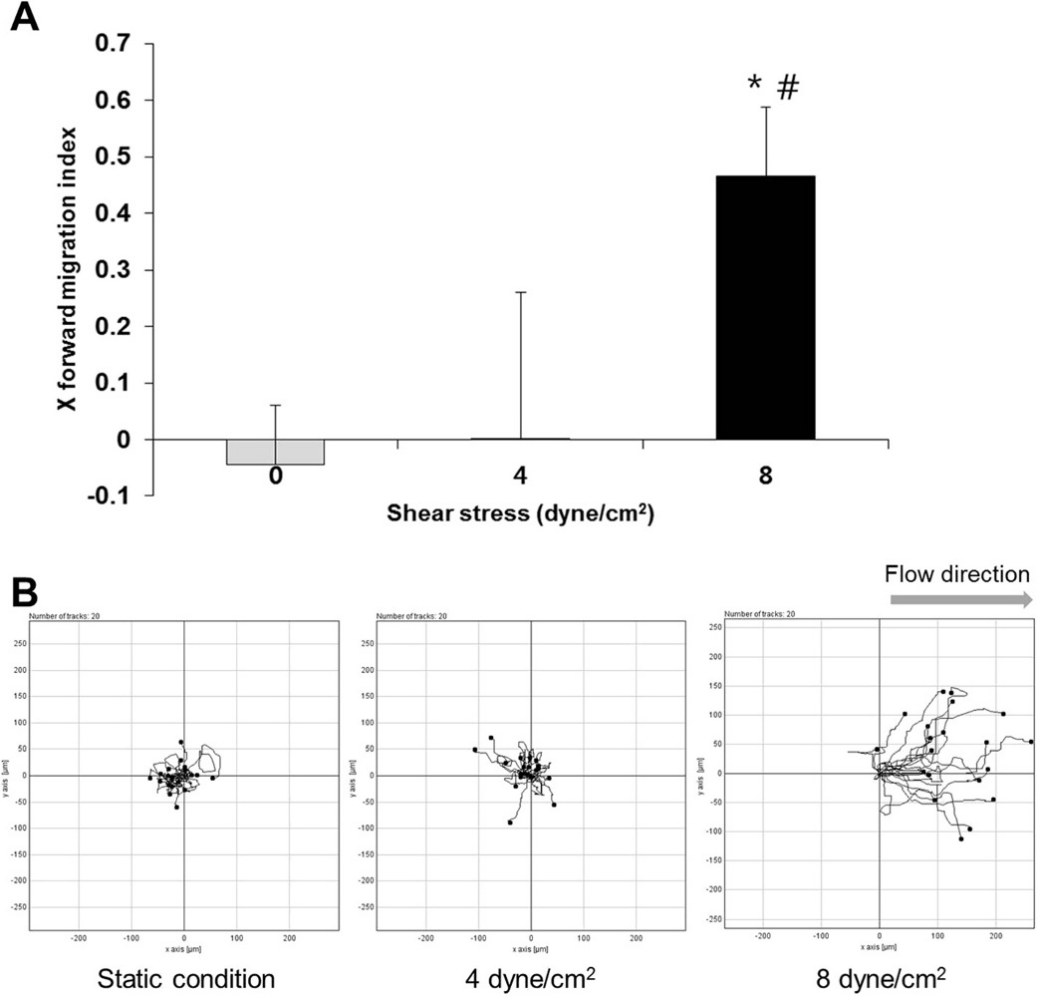
**The directional migration of HUVEC in individual cell migration model. (A)** X_FMI_ of HUVEC. **(B)** Trajectories of HUVEC. During 7 hours all cells were assumed to originate at (0,0) under static conditions and fluid shear stress (4 dyne/cm^2^, 8 dyne/cm^2^). The movement of 20 cells is shown.

When HUVEC were seeded randomly and nonconfluently, they did not tend to move in the direction of flow under static condition and 4 dyne/cm^2^. X_FMI_ between static condition and 4 dyne/cm^2^ was not significantly different (Figure 4A). But when HUVEC were subjected to shear stress at 8 dyne/cm^2^, they started moving in the direction of flow (Figure 4B). X_FMI_ at 8 dyne/cm^2^ moved significantly in the direction of flow than in the absence of flow.

### Actin cytoskeleton alignment

HUVEC were kept as static controls and subjected to a shear stress of 8 dyne/cm^2^ for 30 minutes and 5 hours with the direction of flow from the left to the right. Cells were then fixed and immunostained with Alexa (488)-conjugated phalloidin.

**Figure 5:**
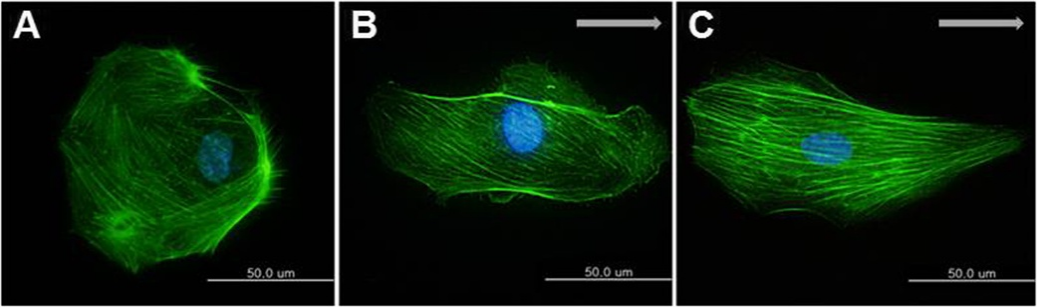
**Immunostaining of actin cytoskeleton.** Actin cytoskeleton was stained with Alexa (488)-conjugated phalloidin (green) under static and flow condition. **(A)** Under static condition **(B)** Shear stress of 8 dyne/cm^2^ was applied for 30 minutes **(C)** Shear stress of 8 dyne/cm^2^ was applied for 5 hours. Flow direction is marked by arrows. Scale bar = 50 μm.

Fluorescent images of actin cytoskeleton were shown in Figure 5A for statically cultured cells and in Figure 5B, Figure 5C for sheared cells. Under static condition (no flow), endothelial cells showed a cobblestone structure with a quite rounded shape. (Figure 5A) Also actin cytoskeleton formed without preferred direction. Figure 5B showed the effect of flow direction on morphological responses of HUVEC at shear stress of 8 dyne/cm^2^ for 5 hours. HUVEC under the flow conditions remodeled their actin cytoskeleton in the direction of applied flow (Figure 5C). Previously many studies have shown that shear stress induces lamellipodial protrusion and focal adhesions (FAs) formation in the flow direction [14, 15]. This could result in EC migration.

### HUVEC migration into scaffold in response of flow

**Figure 6:**
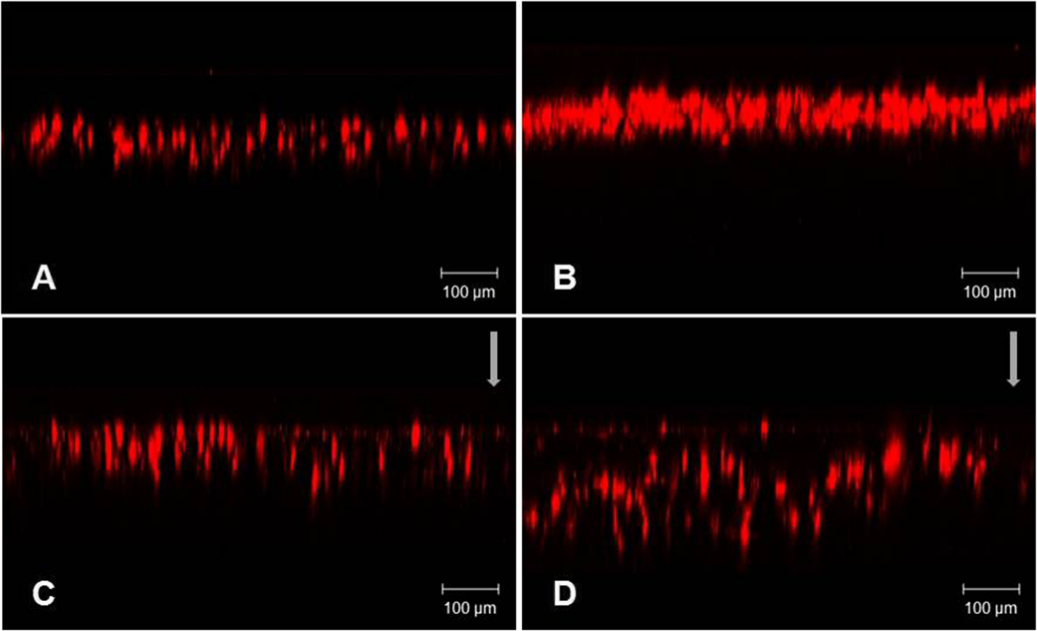
**Cross sectional area of confocal image for cell invasion into scaffold. (A)** Under static condition for 12 hours **(B)** Under static condition for 24 hours **(C)** Under flow condition for 12 hours **(D)** Under flow condition for 24 hours. Flow direction is marked by arrows. Scale bar = 100 μm.

The flow perfusion system was set up for effective cell seeding into the 3D scaffold in the based on the previous data. Microfibrous scaffolds of weigh 80 mg were placed in each 24 well tissue-culture plate. 24 hours after cell seeing onto scaffolds and attachments, cells were cultured under static and flow condition. In results, under static condition, cells could not invade into microfibrous scaffold. Thus, cell tended to remain in the upper part (Figure 6A). Even though cells were cultured for longer time, they did not invade into the scaffold (Figure 6B). On the other hand, under flow condition, cells penetrated into scaffolds more than at the static condition (Figure 6C and D).

## Discussion

In this study, we confirmed the possibility to observe cell migration under static and flow condition using the parallel plate chamber system and to analyze the cell migration speed, directionality and X_FMI_.

For the individual cell migration model, endothelial cells were sparsely plated on coverslips at 30% confluency. This model is appropriate for observing single cell migration. The application of shear stress significantly increased the cell migration speed and cells moved more straightly under flow condition compared to under static condition. The process of adaptation of sparse endothelial cells to shear stress can be divided into two stages regulated by RhoA, Rac1, and Cdc42 [15]. The Rho GTPases Rho, Rac and Cdc42 play several roles in migrating endothelial cells [16, 17]. In the first stage, an increase in RhoA activity leads to the formation of stress fibers and cell contraction. This allows cells to elongate efficiently in the direction of shear stress. This elongation involves directional spreading via protrusion at the front of the cells, and is mediated by Rac and Cdc42 activation. Importantly, RhoA activity is down-regulated at this stage to allow optimal extension of protrusions. Later, RhoA activity increases, and Rho with Rac is required to maintain polarized migration in the direction of shear stress [18].

It was shown in this research that endothelial cells experienced morphological changes when subjected to shear stress. Under static conditions, the cells were characterized by round shape like cobblestone. However, an alteration in shape happened when a shear stress was applied. After the shear stress at 8 dyne/cm^2^ for 5 hours, actin cytoskeleton looked more aligned in the direction of applied flow. Endothelial response of alignment and actin stress fiber induction in the direction of flow is dependent on intracellular calcium [19]. This result explained that actin cytoskeleton did not align in the direction of flow. Shear stress stimulated an increase of intracellular calcium. At first, calcium is released from compartments within the cell, and then later ion channels located on the cell membrane are opened allowing extracellular calcium to input [20]. As a result, signaling pathways associated with calcium may have function in the actin organization changes.

The flow perfusion system was established for effective cell seeding into the 3D scaffold. After cell seeding onto scaffolds and attachments, cells were cultured under static and flow condition. Under static condition, the result showed the cells located in the upper part. They could not enter the microfibrous scaffold. Although the cells were cultured for a longer time, they did not invade into the scaffold. However, when flow was applied to the scaffold, cells penetrated into it more than at the static condition.

Technical problems in cell seeding are produced by the complex structure of the scaffold [21, 22] and insufficient migration into the scaffolds caused by pore size and material, which prolongs the culture period because of the shortage of initially seeded cells [4]. Therefore, a lot of effective seeding methods have been investigated [23] using different scaffolds such as meshes [24] and sponges or mixing conditions that is in static or mixed cultures [25]. In this study, we observed cells under flow condition entered into scaffolds more than under the static condition. This flow perfusion system may help to improve seeding efficiency and provide the possibility about the uniform cell distribution throughout the matrix.

## Conclusions

Shear stress was applied to endothelial cells using the parallel plate chamber and cell movements was characterized by image analysis program that measured the cell migration speed, directionality and X forward migration index. These findings help us to predict the tendency of the cell migration *in vivo* under flow condition based on these *in vitro* results. It may suggest stent designs or the structure of artificial blood vessels for proper re-endothelialization resulting from appropriate ECs migration.

Also we achieved that cells were allowed to migrate into scaffold using the flow perfusion system. This may provide an effective and helpful cell seeding technique to achieve the homogeneous 3D tissue-like structure.
